# Selective porous gates made from colloidal silica nanoparticles

**DOI:** 10.3762/bjnano.6.215

**Published:** 2015-11-04

**Authors:** Roberto Nisticò, Paola Avetta, Paola Calza, Debora Fabbri, Giuliana Magnacca, Dominique Scalarone

**Affiliations:** 1 University of Torino, Department of Chemistry, Via P. Giuria 7, 10125 Torino, Italy; 2 NIS Research Centre, Via P. Giuria 7, 10125 Torino, Italy

**Keywords:** block copolymers, coatings, colloidal nanoparticles, silica, sol–gel synthesis

## Abstract

Highly selective porous films were prepared by spin-coating deposition of colloidal silica nanoparticles on an appropriate macroporous substrate. Silica nanoparticles very homogenous in size were obtained by sol–gel reaction of a metal oxide silica precursor, tetraethyl orthosilicate (TEOS), and using polystyrene-*block*-poly(ethylene oxide) (PS-*b*-PEO) copolymers as soft-templating agents. Nanoparticles synthesis was carried out in a mixed solvent system. After spin-coating onto a macroporous silicon nitride support, silica nanoparticles were calcined under controlled conditions. An organized nanoporous layer was obtained characterized by a depth filter-like structure with internal porosity due to interparticle voids. Permeability and size-selectivity were studied by monitoring the diffusion of probe molecules under standard conditions and under the application of an external stimulus (i.e., electric field). Promising results were obtained, suggesting possible applications of these nanoporous films as selective gates for controlled transport of chemical species in solution.

## Introduction

The development of smart nanoporous devices for the separation of chemical species, ions and biomolecules in solution is a field of increasing interest for researchers involved in microfiltration and separation science [1–7]. In this topic, it is important to remind that microfiltration is one of the oldest processes optimized since the dawn of membrane technology in the 1920s, mostly used for separation of bacteria from water [8]. In the following years, microfiltration devices have found application in several technological fields: water treatments, food industry, biotechnology, electronics and microfluidics [9–13]. Recently, it has been stated that microfiltration devices account for almost half of the whole membrane market [14].

Two different kinds of membranes for microfiltration have been developed over the years: namely screen-filters and depth-filters [8]. Screen-filters, characterized by having well-ordered straight pores, realize the separation by a sieving mechanism based only on pore size: molecules and/or particles smaller than the pore diameter pass easily through the porous membrane, whereas species larger than the pore diameters are retained. They can be obtained by lithographic techniques or templating approaches [15–16]. Depth-filters are characterized by having a tortuous disordered porous network. Even if the real mechanism of separation is not clear yet, particles are generally supposed to be retrained within the filter bulk thorough adsorption and mechanical entrapments [8].

A step forward in the preparation of microfiltration devices was realized by the surface functionalization of macroporous supports or membranes by nanoporous functional coatings [17–20]: the macroporous, permeable supports, in fact, can provide mechanical strength to the thinner functional coatings on top, thus becoming resistant selective gates [4].

Microsieve membranes are very thin flat-sheet devices with a well-ordered porous organization. They can be made of different materials, either inorganic (such as silicon or silicon nitride) or organic (such as polysulfone or polyethersulfone). Silicon nitride (Si_3_N_4_) inorganic microsieves are mainly used in the semiconductor industry [21], even though recently they are finding application in the clarification of milk, beers and juices as well as in biotechnology for the separation of bacteria and/or blood cells [7,22].

Silicon nitride microsieves with hexagonally ordered pores were also employed as substrate for MCM-48 silica films, giving promising results [4,23–24]. The sol–gel polymerization process is a key procedure for the bottom-up synthesis of nano- and mesoporous silica films and in the literature there are several reviews focusing on this field [25–26]. Conventional procedures for the synthesis of mesoporous silica involve the use of amphiphilic templates [27–30]. Either low molecular weight surfactants or polymers have been used as structure-directing agents in the preparation of organic–inorganic hybrid solutions and they have proved to generate a variety of well-ordered materials by self-assembling processes [31–35].

Here, we describe the synthesis, deposition and physicochemical characterization of silica coatings, obtained by spin-coating deposition of soft-templated silica colloidal nanoparticles onto commercial Si_3_N_4_ microsieves for membrane applications. Moreover, permeability and size-selectivity were studied by monitoring the diffusion of different probe molecules under standard conditions and under the application of an electric field as external stimulus. Selected probe molecules were the cationic dye methylene blue (MB, molecular weight (*M*_W_) = 320 Da) and the cationic protein ribonuclease A (RNAse, *M*_W_ = 13700 Da).

## Results and Discussion

### Synthesis, preparation and physicochemical characterization of the colloidal silica nanoparticles and mesoporous coatings

**Figure 1:**
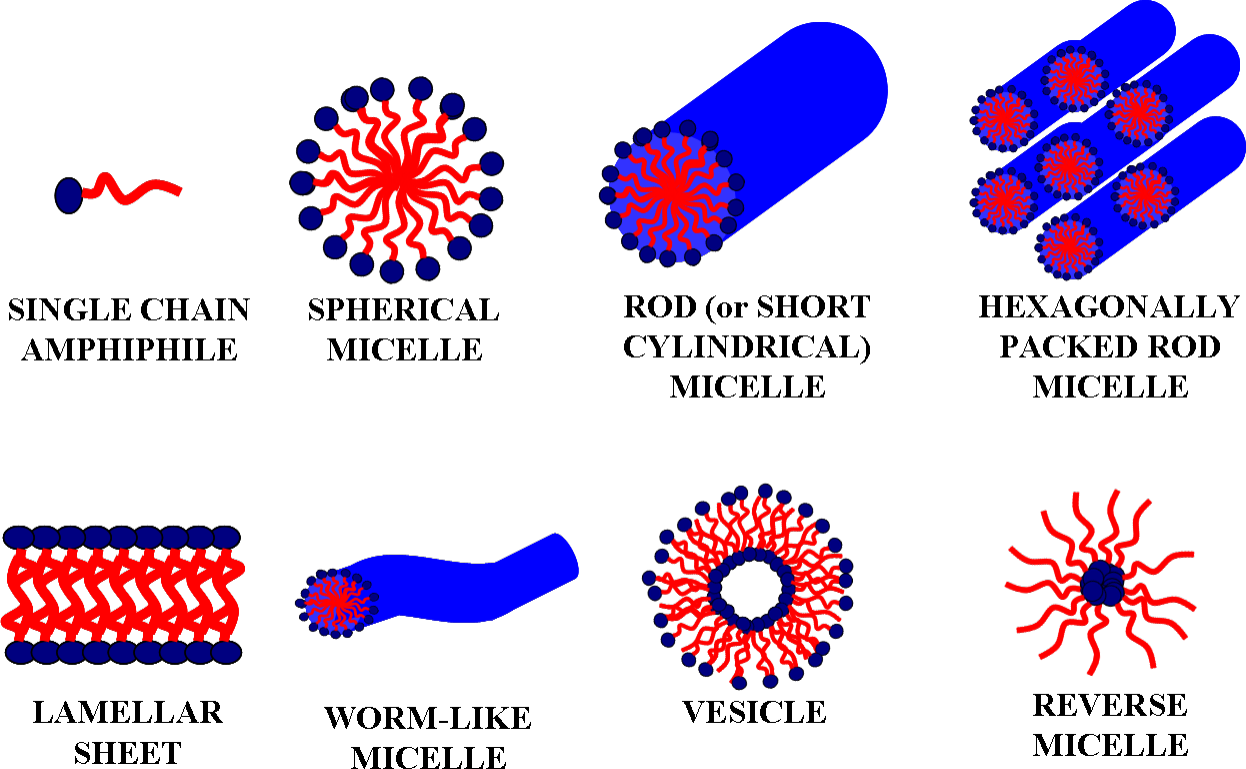
Schematic representation of different micellar architectures. Hydrophilic polar heads are indicated in blue, whereas hydrophobic non-polar tails are drawn in red.

Amphiphilic block copolymers in solution are able to form various types of aggregates, such as micelles and vesicles that can be employed to build novel nanomaterials [36–37]. Figure 1 reports the possible supramolecular organizations of amphiphiles when dissolved in solution. In particular, by changing the ratio between the silica precursor (i.e., tetraethyl orthosilicate, TEOS) and the soft-templating agent (block copolymer), different architectures of the final oxidic material can be achieved [27]. The driving force for self-assembling is the thermodynamic incompatibility of the different blocks in the polymeric chains, which brings them to spontaneously segregate in well-defined nanostructures. Therefore, when block copolymers are mixed to solvents which are selective for one of the blocks, polymer chains spontaneously aggregate into micelles having different architectures (i.e., spheres, rods, tubes, lamellae) and degree of order depending on the physicochemical properties of the block copolymer [38]. Next to the widespread spherical and short cylindrical (rod-like) micellar systems, also other types of supramolecular organizations were found, like lamellar sheets [39], worm-like systems [27] and vesicles [38]. When reverse micellization takes place, reverse micelles can work as nanoreactors [40] and used to produce nanoparticles.

**Figure 2:**
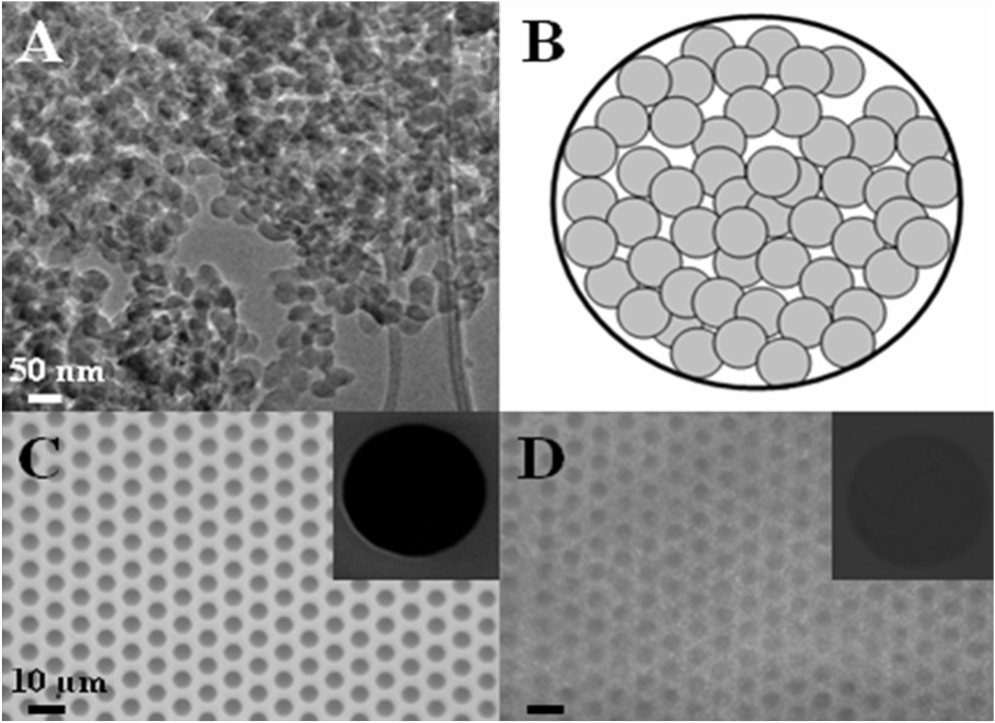
TEM micrograph of 75TEOS/25PS_308_-*b*-PEO_250_ silica nanoparticles (A), schematic top-view of a depth-filter functionalized 5 μm pore (B), optical micrograph of a Si_3_N_4_ microsieve before (C) and after (D) fuctionalization with silica nanoparticles. Insets in pictures C and D show the SEM micrograph of a single microsieve pore collected at high magnification.

Basing on our results, the reverse micellization regime definitively establishes with a TEOS/block copolymer weight ratio of 75/25 and the corresponding samples, obtained after calcination, appear as aggregates of individual silica nanoparticles with an average diameter of 25–30 nm (Figure 2A).

Once the colloidal solution is deposited via spin-coating onto the macroporous support and then calcined to remove organic moieties, colloidal silica nanoparticles aggregates, forming a layer covering the macroporous support. Figure 2B represents a schematic top-view of a single macropore (diameter of 5 μm) functionalized with nanoparticles in a depth-filter arrangement. The tortuous porosity is due to the tiny voids between the nanoparticles (interparticles voids) forming a disordered porous network. Figure 2C and 2D represent the Si_3_N_4_ microsieve surface before and after functionalization with the colloidal silica particles. The coating seems to be homogeneous, as confirmed by insets showing micrographs of the individual pores collected at higher magnification.

**Figure 3:**
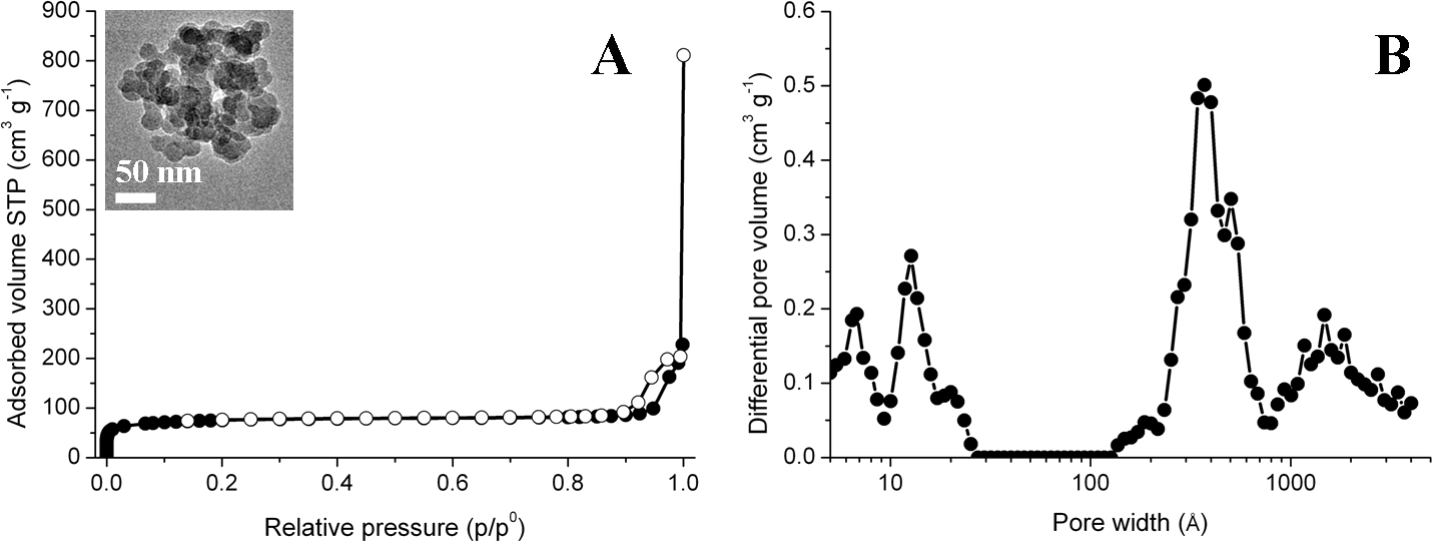
N_2_ adsorption-desorption isotherm at 77 K (A) and DFT pore size distribution curve of 75TEOS/25PS_308_-*b*-PEO_250_ silica powder (B). Dark symbols refer to the adsorption branch of the isotherm, empty symbols to the desorption branch. Inset shows the TEM micrograph of the powdery samples obtained by solvent-casting deposition.

In order to evaluate the porosity of such depth-filter coatings, thicker samples of large weight were prepared by solvent-casting and N_2_ adsorption/desorption gas-volumetric analyses were performed. TEM measurements confirmed that the casting procedure adopted for this preparation provided a morphology similar to spin-coated materials (see inset in Figure 3A). The N_2_ gas-volumetric isotherm shown in Figure 3A is of the IV type, with a small hysteresis loop of H2 type (from IUPAC classification) in the relative pressure range 0.9–1, next to the condensation limit. The BET surface area is of ca. 260 m^2^ g^−1^ and the DFT pore size distribution curve (Figure 3B) indicates a complex pore size distribution. In detail, pores present a bimodal distribution, with the presence of meso/macroporosity in the range 15–200 nm, probably due to interparticle voids (i.e., depth-filter porosity), together with a certain degree of microporosity in the range 1–2 nm, probably due to intraparticle voids generated from the elimination of poly(ethylene-oxide) moieties during the calcination step [41].

### Transport testing of the functional coating

**Figure 4:**
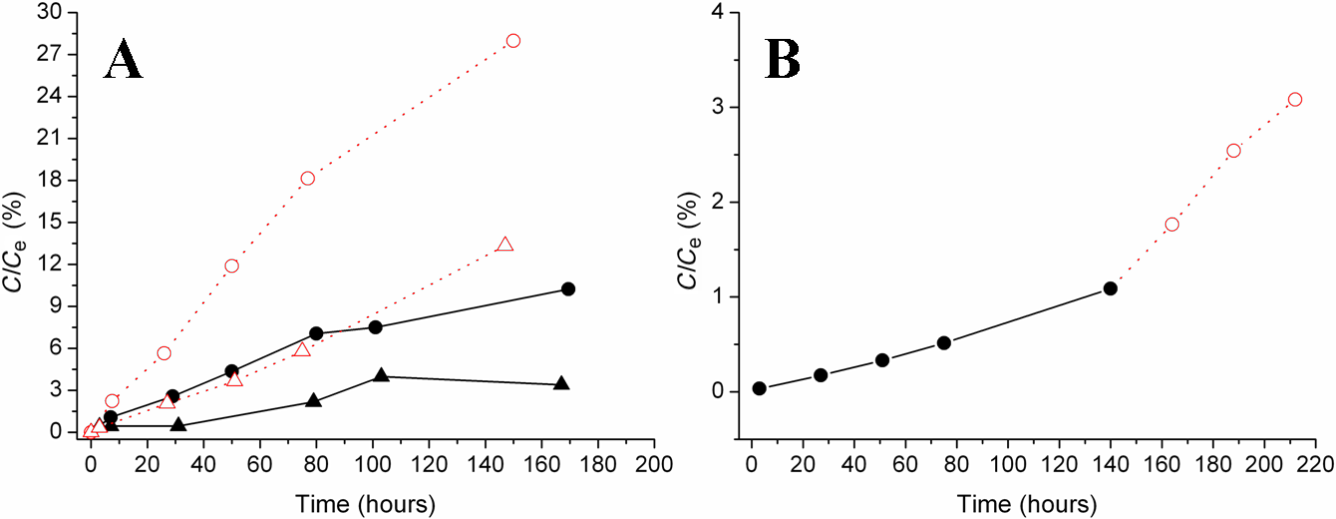
Concentration of probe molecules transported through the silica nanoparticle-functionalized microsieve. Section A: diffusion (black symbols, black solid lines) and migration (red symbols, red dotted lines) of MB (circle) and RNAse (triangle). Section B: diffusion (black circle, black solid line) followed by migration after 140 h (red circle, red dotted line) of MB in a mixed solution containing both MB and RNAse.

UV–vis spectroscopy was used to study the diffusion of two positively charged chemical probes (i.e., MB and RNAse) through the composite membrane. The hydrodynamic radius of MB is 0.5 nm [42], whereas the molar diameter of RNAse is approximately 3.8 nm [43]. According to these values, both probes should cross the depth-filter device whose porosity is in the range of 15–200 nm. In Figure 4A the values of percentage ratio between the effective concentration (*C*) of probe molecules passed through the membrane and the concentration at the equilibrium (*C*_e_) is plotted as a function of time. The resulting diffusion curves demonstrate that dye molecules cross the membrane more easily than the protein which is partially blocked: at the time value of 167 h the diffusion of MB is ca. 10%, whereas for RNAse it is significantly lower, ca. 3%. These trends evidence a steric selectivity of the silica membrane. Interestingly, the functionalized membranes can be regenerated and reused by gently washing them with 2-propanol. Transport tests carried out using a regenerated membrane gave diffusion curves that are very similar to those reported in Figure 4A, thus proving that the proposed composite membranes are not damaged by the cleaning treatment and can be reused. This means that the interactions between the silica surface and probe molecules are weak and labile interactions that can be broken by mild treatments. Also because of this, the membrane selectivity is here mainly attributed to steric effects, while specific interactions and adsorption phenomena have been ignored.

In addition, the migration properties of the membrane were tested by applying an electric field as external stimulus. It was found that it is possible to accelerate the passage of both probes trough the membrane without any loss in selectivity. In fact, at the time value of 147 h the migration of MB is ca. 28%, showing an increase of 18% with respect to the plain diffusion, whereas for RNAse migration is ca. 13%, corresponding to an increase of 10% with respect to the diffusion.

By making a comparison between diffusion and migration conditions, two main aspects deserve to be stressed. First, the application of an electric field increases the transport rate of the two species, which are both positively charged at the pH condition of the experiment, but it does not affect the membrane selectivity. This will result in a reduction of the time required for the separation. Second, the different increase in the transport rate of MB and RNAse under the migration regime proves that transport is affected by both the probe size and the effective charge. In fact, after 147 h MB transport passed from 10% to 28% with an increase of ca. 180%, whereas RNAse passed from 3% to 13% with an increase of ca. 333%.

Moreover, in order to clarify the effect of RNAse on the MB transport a further experiment was carried out. A mixed solution containing both MB and RNAse was prepared and the transport of MB through the membrane was evaluated examining the intensity of the signal at 664 nm. As reported in Figure 4B, the amount of MB diffused in the presence of RNAse at the time value of 140 h is ca. 1%, whereas for the neat MB solution it is ca. 9%. Furthermore, it can be observed that the application of an electric field causes a sharp increase of the amount of MB passing through the membrane. However, the amount of dye which migrates in the receiving cell is still lower if compared to the experiments carried out in the presence of MB alone. In general, these data suggest that the MB transport kinetics in the mixed solution were slowed down by the presence of RNAse.

Further studies are currently in progress in order to elucidate if this behavior has to be ascribed to specific interactions between MB and RNAse or to membrane fouling due to preferential interactions between RNAse and the Si_3_N_4_ surface.

## Conclusion

Large-mesopore silica thin films were prepared via soft-templating by using PS-*b*-PEO block copolymer micelles and were characterized in order to assess their applicability as selective gates for controlled dosing and transport of chemical species in aqueous solution.

The development of mesoporous silica membranes with depth-filter porous organization opens new perspectives in the production of miniaturized devices for separation processes and dosing of chemicals. In this study, the surface functionalization of silicon nitride commercial microsieves by means of colloidal silica nanoparticles has been proposed as a novel strategy to fabricate composite membranes for microfluidic devices.

The permeability and size-selectivity of the functional microsieves we have prepared were studied by monitoring the diffusion of probe molecules with different molecular weight (i.e., methylene blue and ribonuclease A) under standard conditions and under the application of an external stimulus (i.e., electric field). Promising results have been obtained, suggesting possible applications of these mesoporous films as selective gates for controlled transport of chemical species in solution.

## Experimental

### Synthesis and preparation of coatings from colloidal silica nanoparticles

Colloidal silica nanoparticles were synthesized by sol–gel reaction of tetraethyl orthosilicate (TEOS, 99.0%, Aldrich) in ethanol (95.0%, Carlo Erba Reagents) under acidic conditions (HCl 37 wt %, Fluka Chemika), with a TEOS/HCl molar ratio of 3.5 and in the presence of PS_308_-*b*-PEO_250_ (*M*_n_ = 32,000-*b*-11,000, Polymer Source Inc., Dorval, Canada) as structure directing agent. Benzene (≥99.7%, Riedel-de-Haën) was used as a solvent to solubilize the block copolymer. All chemicals were used without further purifications.

Copolymer benzene solutions (1 wt %) were prepared and let stirring until complete dissolution of the copolymer. Micellar solutions were obtained by adding the proper amount of sol–gel solution, as reported in previous studies [27]. In particular, the TEOS/PS_308_-*b*-PEO_250_ weight ratio was fixed to 75/25.

**Figure 5:**
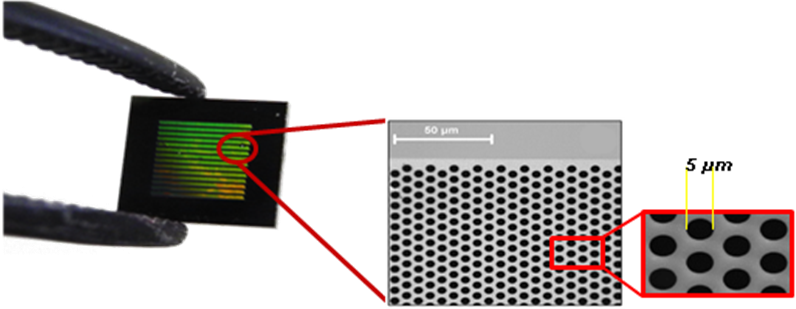
Macroporous commercial silicon nitride microsives purchased from Aquamarijn Micro Filtration BV (Zutphen, Netherlands), having on top a Si_3_N_4_ membrane (5 mm × 5 mm × 0.6 mm) with macropores arranged in a hexagonal setting and diameter of 5 μm.

The final solution was spin coated at 1000 rpm for 20 s, using a 8” Desk-top Precision Spin Coating System, model P-6708D vs. 2.0, both onto mica sheets and on commercial silicon microsieves purchased from Aquamarijn Micro Filtration BV (Zutphen, Netherlands). Silicon microsieves have on top a 5 mm × 5 mm × 0.6 mm Si_3_N_4_ membrane with macropores having a diameter of 5 μm and arranged in a hexagonal setting (Figure 5). After deposition, functionalized hybrid materials were dried in a hood at RT for at least 12 h in order to reach complete evaporation of solvents. Hybrid coatings were then transformed into colloidal silica nanoparticle layers by thermal treatments in a furnace under air atmosphere (400 °C for 2 h, ramp of 2 °C/min).

### Synthesis and preparation of the colloidal silica powder

The same reactants described above were used to prepare silica powders for N_2_ gas-volumetric adsorption at 77 K. In this case the sol–gel solutions were deposited onto glass Petri dishes, the solvent was evaporated, the hybrid films gently scratched and then calcined using the same conditions applied for the coating preparation (400 °C for 2 h, ramp of 2 °C/min). Silica powders thus obtained were analyzed by HRTEM to verify that their morphology was identical to that of thin spin-coated films obtained from the same micellar solution.

### Physicochemical characterization

Transmission electron microscopy (HRTEM) was used to evaluate the morphology of colloidal silica nanoparticle coatings after the removal of the templates. Micrographs were obtained by using a JEOL JEM 2010 instrument (300 kV) equipped with a LaB_6_ filament. For the specimen preparation a few drops of water were poured on the supported silica layer. After few seconds the surface was gently scratched and the functionalized layer separated from the support. Fragments were then transferred onto holed carbon coated copper grids by lifting the grids onto the water layer. Integrity and large-scale homogeneity of the membranes prepared on silicon microsieves were assessed by a Leica DM2005 optical microscope equipped with a digital camera for image acquisition and by scanning electron microscopy (SEM) [42].

SEM analyses were carried out using a ZEISS EVO 50 XVP with LaB_6_ source, equipped with detectors for secondary electrons collection and EDS probe for elemental analyses.

N_2_ adsorption-desorption experiments were carried out by means of ASAP 2020 instrument (Micromeritics) in order to determine specific surface area (BET model) [44] and porosity (DFT method) [27,45] of samples. The density functional theory (DFT) model for slit pores with low regularization was applied on the adsorption branch of the isotherm in order to examine simultaneously both micro- and mesoporosity of samples. The analyses were performed on powdery samples (ca. 100 mg) outgassed for several hours at 300 °C in vacuo (residual pressure 10^−2^ mbar) to ensure complete removal of atmospheric contaminants from surface and pores.

### Transport tests

Transport tests were carried out following the same procedure already described elsewhere [42]. A homemade side-by-side diffusion cell was used, consisting of two half-cells in Pyrex physically separated by the porous membrane, fixed by two silicone seals covered with Teflon and held together with a metal clamp. The sealing of the system was evaluated with diffusion tests performed in the presence of a metallic diaphragm in place of the membrane. TOC analysis excluded the release of organic impurities from the device. TOC analysis were carried out with a Shimadzu TOC-VCSH Total Organic Carbon Analyzer, equipped with an ASI-V autosampler and fed with zero-grade air (Sapio, Italy).

Solutions of target molecules, i.e., methylene blue (MB in the following, Sigma Aldrich, 5.0 × 10^−4^ mol L^−1^, water solution) and/or ribonuclease A from bovine pancreas (RNAse in the following, Sigma Aldrich, 1.0 wt %, water solution) [46], were put in the donor cell and the passage through the membrane was evaluated by means of UV–vis spectrophotometric analysis (UV–vis spectrometer Lambda 25 Perkin–Elmer) performed on the solution in the receptor cell (filled with water). Diffusion tests were carried out under continuous stirring in both cells at acid pH (thus both probes are positively charged). Spectrophotometric determination of target molecules was done at 664 nm for MB and at 277 nm for RNAse. Quantification was performed with external calibration curves (R^2^ ≥ 0.9999 for MB and R^2^ ≥ 0.9998 for RNAse, see Figures S1 and S2 in Supporting Information File 1). Additionally, some tests were performed applying an external stimulus (i.e., electric field) by using a battery of 9 V connected with two graphite electrodes immersed in the two half-cells, with the positive electrode in the donor cell and the negative one in the receptor cell. Reported transport tests are averages of two replicas.

## Supporting Information

File 1 External calibration curves performed by UV–vis spectroscopy.
