# Supplementary material for: Selective porous gates made from colloidal silica nanoparticles
Source: Beilstein J Nanotechnol. 2015 Nov 4;6:2105–12. doi: 10.3762/bjnano.6.215 (PMC4660950; doi:10.3762/bjnano.6.215)
Supplement: File 1 — External calibration curves performed by UV–vis spectroscopy. [file Beilstein_J_Nanotechnol-06-2105-s001.pdf]

Supporting Information

for

Selective porous gates made from colloidal silica nanoparticles

Roberto Nisticò*[1], Paola Avetta[1], Paola Calza[1,2], Debora Fabbri[1], Giuliana Magnacca[1,2] and Dominique Scalarone[1,2]

Address: [1]University of Torino, Department of Chemistry, Via P. Giuria 7, 10125 Torino, Italy and [2]NIS Research Centre, Via P. Giuria 7, 10125 Torino, Italy

Email: Roberto Nisticò - roberto.nistico@unito.it

* Corresponding author

Supporting Information

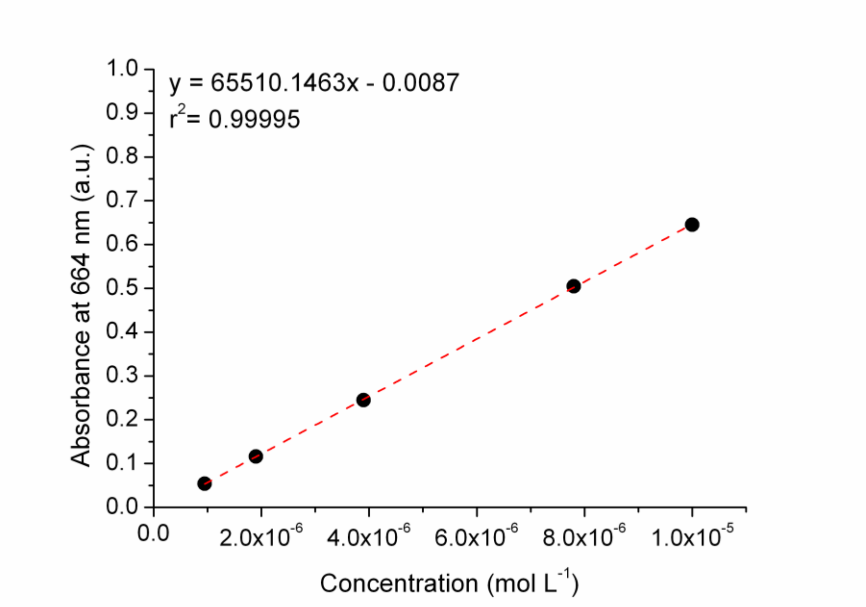

Figure S1: Calibration curve of MB determined at 664 nm: Absorbance as a function of MB concentration. Reliability of regressions: R^2 = 0.99995.

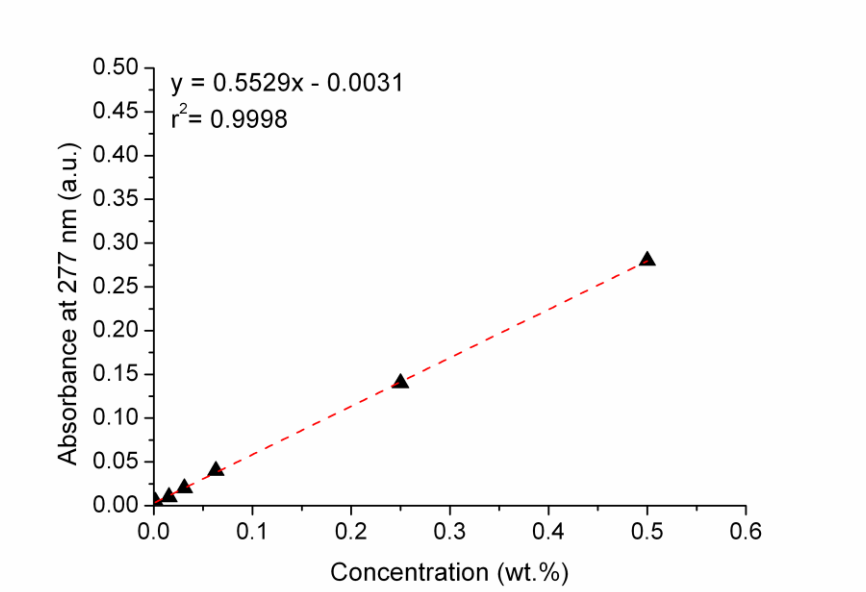

Figure S2: Calibration curve of RNAse determined at 277 nm: Absorbance as a function of RNAse concentration. Reliability of regressions: R^2 = 0.9998.
